# Effects of a Person-Centered eHealth Intervention for Patients on Sick Leave Due to Common Mental Disorders (PROMISE Study): Open Randomized Controlled Trial

**DOI:** 10.2196/30966

**Published:** 2022-03-15

**Authors:** Matilda Cederberg, Sara Alsén, Lilas Ali, Inger Ekman, Kristina Glise, Ingibjörg H Jonsdottir, Hanna Gyllensten, Karl Swedberg, Andreas Fors

**Affiliations:** 1 Institute of Health and Care Sciences Sahlgrenska Academy University of Gothenburg Gothenburg Sweden; 2 University of Gothenburg Centre for Person-Centred Care (GPCC) Sahlgrenska Academy University of Gothenburg Gothenburg Sweden; 3 Department of Psychiatry Sahlgrenska University Hospital Gothenburg Sweden; 4 Department of Internal Medicine and Geriatrics Sahlgrenska University Hospital Östra Gothenburg Sweden; 5 The Institute of Stress Medicine Region Västra Götaland Gothenburg Sweden; 6 School of Public Health and Community Medicine Sahlgrenska Academy University of Gothenburg Gothenburg Sweden; 7 Department of Molecular and Clinical Medicine Sahlgrenska Academy University of Gothenburg Gothenburg Sweden; 8 Research and Development, Primary Health Care Region Västra Götaland Gothenburg Sweden

**Keywords:** depression, anxiety, stress, patient-centered care, person-centered care, telehealth, mHealth, sickness absence, intervention, randomized controlled trial, mobile phone

## Abstract

**Background:**

Sick leave due to common mental disorders (CMDs) is a public health problem in several countries, including Sweden. Given that symptom relief does not necessarily correspond to return to work, health care interventions focusing on factors that have proven important to influence the return to work process, such as self-efficacy, are warranted. Self-efficacy is also a central concept in person-centered care.

**Objective:**

The aim of this study is to evaluate the effects of a person-centered eHealth intervention for patients on sick leave due to CMDs.

**Methods:**

A randomized controlled trial of 209 patients allocated to either a control group (107/209, 51.2%) or an intervention group (102/209, 48.8%) was conducted. The control group received usual care, whereas the intervention group received usual care with the addition of a person-centered eHealth intervention. The intervention was built on person-centered care principles and consisted of telephone support and a web-based platform. The primary outcome was a composite score of changes in general self-efficacy (GSE) and level of sick leave at the 6-month follow-up. An intention-to-treat analysis included all participants, and a per-protocol analysis consisted of those using both the telephone support and the web-based platform.

**Results:**

At the 3-month follow-up, in the intention-to-treat analysis, more patients in the intervention group improved on the composite score than those in the control group (20/102, 19.6%, vs 10/107, 9.3%; odds ratio [OR] 2.37, 95% CI 1.05-5.34; *P*=.04). At the 6-month follow-up, the difference was no longer significant between the groups (31/100, 31%, vs 25/107, 23.4%; OR 1.47, 95% CI 0.80-2.73; *P*=.22). In the per-protocol analysis, a significant difference was observed between the intervention and control groups at the 3-month follow-up (18/85, 21.2%, vs 10/107, 9.3%; OR 2.6, 95% CI 1.13-6.00; *P*=.02) but not at 6 months (30/84, 35.7%, vs 25/107, 23.4%; OR 1.8, 95% CI 0.97-3.43; *P*=.06). Changes in GSE drove the effects in the composite score, but the intervention did not affect the level of sick leave.

**Conclusions:**

A person-centered eHealth intervention for patients on sick leave due to CMDs improved GSE but did not affect the level of sick leave.

**Trial Registration:**

ClinicalTrials.gov NCT03404583; https://clinicaltrials.gov/ct2/show/NCT03404583

## Introduction

### Background

The term common mental disorders (CMDs) is used to describe the most prevalent mental disorders worldwide, such as depression and anxiety [1]. CMDs can also include stress-related disorders comprising; for example, adjustment disorder, reaction to acute stress, and burnout or exhaustion disorder. CMDs are a frequent cause of sick leave episodes, which have a long mean duration and a risk of recurrence, making them a significant issue for many countries and health care systems [2-6]. For the affected individual, sick leave can be a necessary component in the treatment process, but long-term sick leave and relapse may have detrimental effects such as social exclusion and transition to disability pension [7]. In Sweden, the number of sick leave episodes linked to CMDs has increased during the past decade, especially those caused by adjustment disorders and reactions to severe stress. In 2016, almost half of all ongoing sick leave episodes in Sweden were linked to CMDs [8]. Most CMDs are treated in primary care [9], and according to the Swedish national guidelines, treatment for depression and anxiety should consist of medication or cognitive behavioral therapy or both [10]. There is no such consensus on treatment for stress-related disorders [11,12]. Internet interventions have proven to be a viable alternative to face-to-face treatments in patients with CMDs [13-16]. Because of the high accessibility and direct involvement of the patient, internet interventions may also enhance self-management [17]. To date, very few studies have evaluated interventions using eHealth alternatives for CMDs that specifically target return to work (RTW) [18].

The associations between illness improvement and sick leave are multiple and complex, given that RTW does not necessarily correspond to symptom relief [19,20]. To influence sick leave and improve RTW among persons on sick leave due to CMDs, interventions that specifically focus on RTW seem to be more successful than clinical interventions alone [21-25].

Self-efficacy and involving the workplace are two factors, modifiable by interventions, whose importance for the RTW process has been confirmed in several studies [25-32]. Self-efficacy is a psychological concept describing an individual’s judgment of their ability to manage challenging situations. Thus, self-efficacy can serve as an important psychological resource contributing to employee adjustment and well-being, reducing vulnerability to stressors, and increasing resilience in coping with adverse events [33-35]. Several studies have shown an association among higher self-efficacy beliefs, shorter time to RTW [29,30], and sustainable RTW [28]. Lagerveld et al [32] broadened the scope by suggesting that not only did higher initial levels of self-efficacy have a prognostic value for RTW, but an increase in self-efficacy during an intervention also predicted a shorter sick leave duration until full RTW.

### Person-Centered Care

Person-centered care (PCC) is an approach within health care based on ethical principles stressing the importance of treating the patient as a person and the cocreation of care through partnerships built on trust, respect, and mutuality [36-40]. In addition, PCC is a clinical pathway of care that adheres to evidence-based practice. Studies evaluating interventions based on PCC have shown positive effects of self-efficacy in various health care settings, targeting various conditions [40]. Furthermore, studies in which PCC has been applied partially [41] or entirely [42] in remote settings have shown positive effects on self-efficacy. Self-efficacy is a key concept in PCC that aims to enhance patients’ confidence in their ability to manage their condition through supportive partnerships [43]. As previous studies based on PCC have shown positive effects on self-efficacy and because of the potential influence of self-efficacy in affecting sick leave, it is warranted to construct and evaluate PCC interventions targeting patients on sick leave due to CMDs. Thus, this study aims to evaluate the effects of a person-centered eHealth intervention for patients on sick leave due to CMDs.

## Methods

### Study Setting

The study took place in a large, socioeconomically diverse city area in western Sweden, and 9 public primary health care centers participated. The intervention and each study procedure were managed remotely. The study was an open randomized controlled trial with 1:1 allocation to either a control group receiving usual care only or an intervention group receiving usual care in conjunction with a person-centered eHealth intervention comprising telephone support and access to a web-based platform. In Sweden, the social insurance system allows 7 days of sick leave (plus an initial qualifying day) without a medical certificate. Employees are usually covered by their employer during the first 14 days of sick leave. After that, benefits can be granted from the State Social Insurance Agency. Because of the COVID-19 pandemic, these regulations have been subject to a few temporary changes. The qualifying day was suspended from March 11, 2020, and employees were financially covered from day 1. From April 2, 2020, a medical certificate was required from the 15th day of illness. As enrollment in the study closed in June 2020, these temporary regulations were valid during the last months of the enrollment period.

### Participants and Recruitment

Patients aged 18-65 years were eligible if they were currently on sick leave due to one of the following conditions in the International Statistical Classification of Diseases and Related Health Problems, Tenth Revision, and diagnosed by a physician: mild to moderate depression (F32 and F33), mild to moderate anxiety disorder (F41), reaction to severe stress, and adjustment disorders (F43, except posttraumatic stress disorder), which include the Swedish diagnosis exhaustion disorder (F43.8A). To reach patients early in their sick leave process, their current sick leave episode should not have exceeded 30 days. Patients were eligible only if they were employed or studying at least part-time during the past 9 months. Only patients with a registered address in Sweden and able to manage the Swedish language were included. Patients were excluded if they had previous sick leave due to depression, anxiety disorders, and stress reactions and disorders exceeding 14 days over the past 3 months. Other exclusion criteria were severe impairments preventing the use of the eHealth intervention, ongoing alcohol or drug abuse, severe disease with an expected survival of <12 months or that could interfere with follow-up, if the intervention was assessed as a burden, or if the patient was participating in a conflicting study.

### Enrollment and Randomization

**Figure 1:**
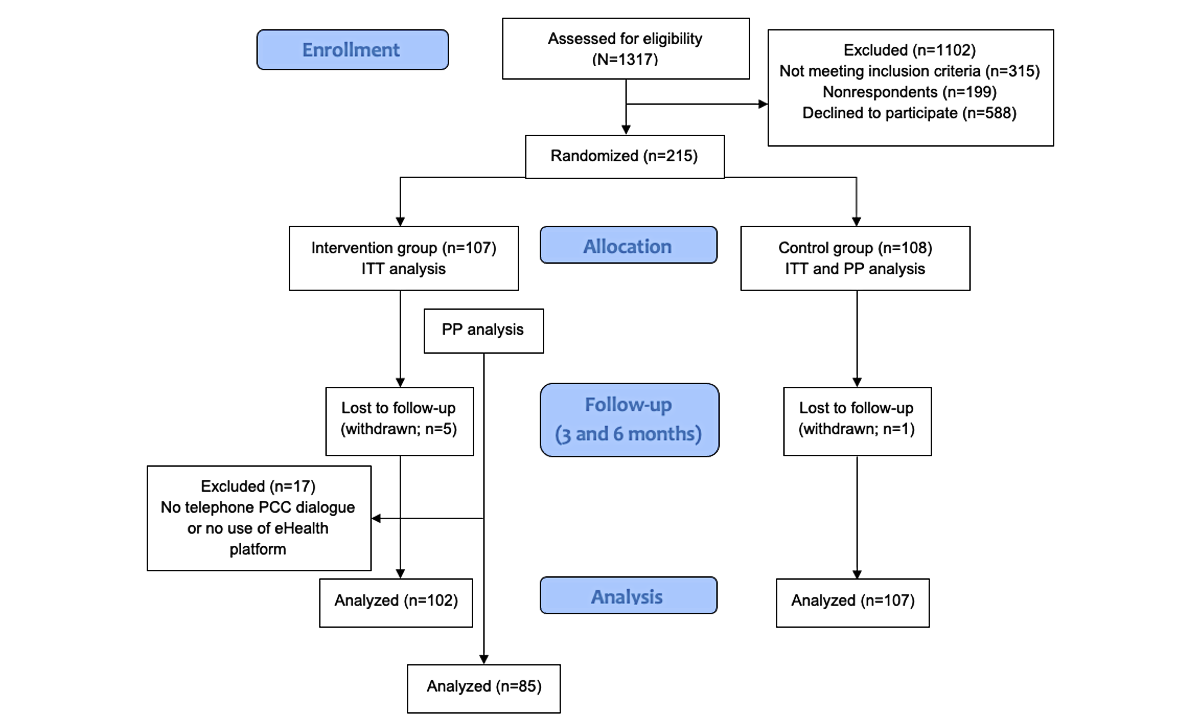
CONSORT (Consolidated Standards of Reporting Trials) diagram. ITT: intention-to-treat; PCC: person-centered care; PP: per-protocol.

Participant recruitment lasted from February 2018 to June 2020. Figure 1 presents a flowchart of the trial. Designated health care professionals (HCPs) consecutively screened the medical records of 9 primary health care centers for eligible participants. Eligible participants were sent an information letter about the study, notifying them that further contact would be made. Next, patients were contacted by telephone or they contacted the HCPs using the instructions provided in the information letter. More information about the study was provided over the telephone. Patients interested in participating were sent a consent form and information about their rights as participants by regular mail. After written consent had been returned by mail to the HCPs, patients were randomized to the control or intervention group. Randomization was based on a computer-generated random list created by a third party and stratified by age (<50 years or ≥50 years) and diagnostic group (1: depression, 2: anxiety, and 3: stress reactions and disorders). After randomization, participants were informed of their study arm.

### Usual Care

Patients on sick leave for CMDs are usually offered an appointment with a physician to follow-up on sick leave and make treatment decisions. Treatment may consist of medication or psychological therapies such as cognitive behavioral therapy [10]. Depending on the services available at each primary health care center, usual care can also include contact with a physiotherapist, rehabilitation coordinator, or occupational therapist, as well as group sessions targeting specific symptoms or problems.

### Intervention

#### Overview

In addition to usual care, the intervention group received PCC through a web-based platform and telephone support during the 6-month intervention period. A detailed description of the intervention has been published elsewhere [44]. The intervention aims to operationalize person-centered ethics by safeguarding the relational aspects of personhood and care according to PCC [39,45]. HCPs from different disciplines (eg, nursing, physiotherapy, and occupational therapy) conducted the intervention in a research setting separated from the primary health care centers. The team of HCPs received a half-day training and education regarding symptoms, treatments, care, and self-care strategies for CMDs led by psychologists and physicians specialized in stress-related mental illness. They also received an introduction to the philosophical perspective of PCC led by researchers in health and care sciences, philosophy, and pedagogics. The team of HCPs also had access to a regularly held forum where they could, together with specialists in PCC, raise questions and share experiences of the practice of PCC stipulated within the context of the intervention.

By offering infrastructure in the form of telephone support and a web-based platform, the intervention was designed to facilitate the cocreation of care and work in partnership between HCPs and patients (and their extended social network if needed) without face-to-face meetings. The intervention design allowed for individual tailoring in terms of content (ie, the personal health plans) and structure (ie, the number, intensity, and form with regard to communication). The patients were encouraged to use the platform’s different functions, but all use was optional and based on the patient’s preferences. Shortly after inclusion, the HCPs called the patients to help them access the web-based platform, described its features, and scheduled a telephone conversation.

#### Telephone Support

In the telephone conversations the patient’s narrative was central and the HCPs were attentive to the patients’ experiences of their current situation and well-being. HCPs asked questions, encouraged narratives, and listened to the patients’ descriptions to understand how they perceived their condition in the context of daily life. There was no ready-made manual for what the conversations would contain; instead, the ideal conversation was for the content to develop gradually alongside the formation of a mutual relationship in which the patient felt heard and respected. In collaboration with the patients, the HCPs identified strengths and resources (eg, by asking how the patients managed challenging situations) and discussed achievable goals for the near future. The patient’s narrative was documented (by the patient or the HCP) in a cocreated health plan. The health plan contained the patient’s experience of their situation, expressions of needs and resources regarding personal capabilities and the surrounding support system, and what health-related changes they wanted to achieve and how to achieve them. The health plan was uploaded to the web-based platform and served as a starting point for future telephone conversations and communication through the platform. It was modified according to the patient’s health process.

#### Web-Based Platform

The web-based platform was built to be nondirective and to enable patients to participate in their recovery and rehabilitation process. The functions of the platform were designed to create options for the patients’ self-management. The patients could assign daily ratings to their well-being and common symptoms on a scale from 1 to 5, where 1 is poor and 5 is excellent (eg, quality of sleep and ability to concentrate). Their answers were visualized in graphs to allow both patients and HCPs to follow trends and changes over time. The patients could invite family members, other health care contacts, or workplace representatives to access the platform page and functions according to the patient’s choice. The platform was intended to act as a mediator to alert patients and their network to a risk of relapse or worsening symptoms in the recovery process. At least once a day (during office hours), HCPs logged on to the platform to search for messages and get an update on patients’ activities. HCPs could take part of the information on the patient platform before a scheduled conversation, and the patients and HCPs could communicate through the platform in a chat-like forum. Moreover, the patients could receive information on CMDs by means of links to different websites. The platform could be accessed from any device with an internet connection and a web browser (computer, smartphone, or iPad). A participatory design, including workshops with HCPs, patient representatives, and system developers, guided the development of the platform and the intervention [46].

### Data Collection

Data included responses to questionnaires sent by letter at baseline and after 3 and 6 months during the intervention period. Data on full- and part-time sick leave (25%, 50%, and 75% of full-time sick leave) were self-reported in the questionnaires. Data on participants’ gender, age, civil status, country of birth, level of education, occupation, and years of work experience were self-reported at baseline. Self-efficacy was assessed using the General Self-Efficacy Scale (GSES) [47], which has a validated Swedish version [48]. The GSES is a self-assessment questionnaire on a person’s general sense of competence in dealing with unforeseen situations and adversities. The tool consists of 10 items, and responses are made on a 4-point scale (1=not at all true, 2=hardly true, 3=moderately true, and 4=exactly true), resulting in a general score of the sum of all items. The total score ranges from 10 to 40, with higher scores indicating a higher sense of general self-efficacy (GSE).

### Primary Outcome

The primary outcome of this study was a composite score of changes in GSE and level of sick leave at the 6-month follow-up [49]. At the 3- and 6-month follow-ups, participants in both arms were classified as follows:

- Participants with reduced sick leave percentage at follow-up compared with baseline and increased GSE scores by ≥5 units were classified as improved.
- Participants with an increased sick leave percentage at follow-up compared with baseline or reduced GSE scores by ≥5 units were classified as deteriorated.
- Participants who had neither deteriorated nor improved were classified as unchanged.

To calculate the primary outcome both study groups were dichotomized into two subgroups: improved versus unchanged or deteriorated. The designated 5-point difference corresponds closely to the reported SD [50,51] and previous research suggesting 5 points to be a threshold for a minimal important change [42,43]. For further details on participants’ trajectories, the composite score was also analyzed without dichotomizing in an ordered categorical version, including all three possible outcomes (improved or unchanged or deteriorated).

### Sample Size

To achieve a power of 80% based on an *α* error of .05, a minimum of 91 participants was required in each study group to detect an improvement in the composite score (20% in the control group and 40% in the intervention group).

### Statistical Methods

Descriptive statistics were used to characterize the study groups. Between-group differences in baseline characteristics were analyzed using the Pearson chi-square test for categorical variables, Fisher exact test for dichotomous variables, and *t* test (independent, 2-tailed) for continuous variables. Binary logistic regression analysis was used to calculate between-group differences in the dichotomous version of the composite score, differences in improvement of ≥5 units on the GSES, and odds ratios (ORs) with 95% CIs. The Mantel-Haenszel chi-square test was applied for the ordered (3 levels) categorical version of the composite score and self-reported sick leave. Between-group differences in changes in GSE scores were analyzed using the Mann–Whitney *U* test. Missing outcome data for GSE in the composite score at the 3- and 6-month follow-ups were imputed using the last observation value carried forward. Sensitivity analyses were performed to assure robustness by excluding patients who reported causes of sick leave other than CMDs at follow-up (n=3) and participants who reported 0% sick leave at baseline (n=6). These participants who reported 0% sick leave at baseline were on sick leave when they gave oral consent to participate, but their sick leave periods had expired once they were randomized. Intention-to-treat (ITT) and per-protocol (PP) analyses were performed. The PP analysis included intervention participants who had at least one telephone conversation with the HCPs leading to a health plan and who had used the web-based platform at least once during the intervention period. The significance level was set at *P*<.05 (2-sided).

### Ethics Approval

Ethics approval was obtained from the regional ethical review board in Gothenburg, Sweden (DNr 497-17, T023‐18, and T526‐18). A CONSORT-EHEALTH (Consolidated Standards of Reporting Trials of Electronic and Mobile Health Applications and Online Telehealth) checklist is presented in Multimedia Appendix 1.

## Results

### Overview

Of the 215 participants, 108 (50.2%) were randomized to the control group and 107 (49.8%) to the intervention group. After 0.9% (1/108) of the participants in the control group and 4.7% (5/107) of the participants in the intervention group withdrew consent, the final sample included 209 participants: 107 (51.2%) in the control group and 102 (48.8%) in the intervention group. Most of the patients were women (175/209, 83.7%), and the mean age was 42.23 (SD 11.45) years. There were no significant differences in demographic characteristics (eg, age, civil status, education level, and GSE) between the control and intervention groups at baseline (Table 1). Nor were there any significant differences between the groups for the level of current sick leave at baseline or current diagnosis. Of the 209 participants, 44 (21.1%) had depression, 31 (14.8%) had anxiety disorders, and 134 (64.1%) had stress disorders. No significant differences were found between the groups according to illness history (previous episodes of depression, stress, anxiety, or sleep disorder) and current medication. The intervention group had a median of 4 (IQR 0-9) telephone conversations, with an average of 32.6 (SD 10.3) minutes per conversation. In the PP analysis, which excluded 16.7% (17/102) of the participants (of these, 14/17, 82% of participants did not use the web-based platform at any time during the intervention and 3/17, 18%, did not use telephone support), the intervention group had a median of 4 (IQR 1-9) telephone conversations, with an average of 32.8 (SD 10.5) minutes per call.

In the intervention group, 97.1% (99/102) of the participants used the telephone support at least once (mean number of conversations 4.05, SD 1.84), 72.5% (74/102) used the function of self-ratings (number of ratings=1415; mean 19.12, SD 27.23), 49% (50/102) used the function of messages (number of messages=180; mean 3.60, SD 4.07), and 11.8% (12/102) invited at least one person to the platform (number of invitees=15).

**Table 1:** Baseline characteristics (N=209).

Characteristics			Control group (n=107)		Intervention group (n=102)		Per-protocol analysis (n=85)
Age (years), mean (SD)			42.2 (11.7)		42.3 (11.2)		42.7 (11.1)
Women, n (%)			93 (87.7)^a^		82 (80.4)		70 (82.4)
General self-efficacy score, mean (SD)			25.9 (6.1)		25.8 (6.4)		25.9 (6.0)
**Civil status, n (%)**							
	Living alone	30 (28)		40 (39.2)		32 (37.6)	
	Married or living with a partner	77 (72)		62 (60.8)		53 (62.4)	
**Country of birth, n (%)**							
	Sweden	91 (85)		89 (87.3)		73 (85.9)	
	Other	16 (15)		13 (12.7)		12 (14.1)	
**Education level^b^, n (%)**							
	Compulsory	7 (6.6)		6 (5.9)		4 (4.7)	
	Secondary school	16 (15.1)		21 (20.8)		17 (20)	
	Vocational college	20 (18.9)		15 (14.9)		11 (12.9)	
	University	63 (59.4)		59 (58.4)		53 (62.4)	
**Current sick leave, n (%)**							
	0	4 (3.7)		2 (2)		2 (2.4)	
	25	3 (2.8)		5 (4.9)		4 (4.7)	
	50	21 (19.6)		30 (29.4)		25 (29.4)	
	75	3 (2.8)		5 (4.9)		4 (4.7)	
	100	76 (71)		60 (58.8)		50 (58.8)	
**Diagnosis (ICD^c^ codes), n (%)**							
	Stress (F43)	69 (64.5)		65 (63.7)		55 (64.7)	
	Depression (F32 and F33)	23 (21.5)		21 (20.6)		18 (21.2)	
	Anxiety (F41)	15 (14)		16 (15.7)		12 (14.1)	
**Illness history, n (%)**							
	Previous stress	29 (27.4)^a^		34 (33.3)		28 (32.9)	
	Previous depression	28 (26.4)^a^		30 (29.4)		24 (28.2)	
	Previous anxiety	33 (31.1)^a^		29 (28.4)		25 (29.4)	
	Previous sleep disorder	15 (14)		17 (16.7)		15 (17.6)	
**Current medication, n (%)**							
	Antidepressant	54 (50.5)		43 (42.2)		37 (43.5)	
	Sedative	49 (46.2)^a^		37 (36.3)		29 (34.1)	
	Sleep medication	26 (24.5)^a^		25 (24.5)		19 (22.4)	

^a^One value missing.

^b^Two values missing.

^c^ICD: International Statistical Classification of Diseases and Related Health Problems.

### Effects

The ITT analysis showed that a higher percentage of the patients in the intervention group improved on the composite score (improved vs deteriorated or unchanged) than those in the control group at the 3-month follow-up (20/102, 19.6%, vs 10/107, 9.3%; OR 2.37, 95% CI 1.05-5.34; *P*=.04). At the 6-month follow-up, the significant difference between the groups did not persist (31/100, 31%, vs 25/107, 23.4%; OR 1.47, 95% CI 0.80-2.73; *P*=.22). At the 3-month follow-up, a significant difference between the control and intervention groups was observed on the ordered (3-level) composite score (*P*=.04). At the 6-month follow-up, this difference was no longer significant (*P*=.15; Table 2).

In the PP analysis, more participants in the intervention group improved on the composite score at the 3-month follow-up than those in the control group (18/85, 21.2%, vs 10/107, 9.3%; OR 2.6, 95% CI 1.13-6.00; *P*=.02). At the 6-month follow-up, no significant difference was detected (30/84, 35.7%, vs 25/107, 23.4%; OR 1.8, 95% CI 0.97-3.43; *P*=.06). At the 3- and 6-month follow-ups, there was a significant difference between the control and intervention groups on the ordered (3-level) composite score (*P*=.009 and *P*=.03, respectively).

A significant effect was found in GSE alone at the 3-month follow-up between the control and intervention groups (*P*=.03) in the ITT analysis and correspondingly for the PP analysis (*P*=.01). At 6 months, there was no significant difference between the groups in the ITT analysis of GSE (*P*=.07), but the difference remained in the PP analysis (*P*=.04; Table 3).

Sick leave alone did not differ between the groups at 3- or 6-month follow-ups, regardless of analysis (ITT or PP; Table 4). At the 3-month follow-up, 49% (41/83) in the control group, 54% (45/84) in the full intervention group, and 55% (41/75) in the intervention group in the PP analysis reported 0% sick leave. At the 6-month follow-up, the corresponding percentages were 70% (67/96) in the control group, 70% (58/83) in the full intervention group, and 72% (55/76) in the intervention group in the PP analysis.

**Table 2:** Composite scores at the 3- and 6-month follow-ups (N=209).

				Control (n=107), n (%)		Intervention (n=102), n (%)		Odds ratio (95% CI)			*P* value			Per-protocol analysis (n=85), n (%)			Odds ratio (95% CI)			*P* value	
**Three months**																					
	**Composite score**								2.37 (1.048-5.340)			.04						2.61 (1.133-5.996)			.02
		Improved	10 (9.3)		20 (19.6)								18 (21.2)								
		Deteriorated or unchanged	97 (90.7)		82 (80.4)								67 (78.8)								
	**Composite score^a^**											.04									.009
		Improved	10 (10)		20 (20.8)								18 (22)								
		Unchanged	76 (76)		67 (79.8)								59 (72)								
		Deteriorated	14 (14)		9 (9.4)								5 (6.1)								
**Six months**																					
	**Composite score^b^**								1.47 (0.795-2.730)			.22						1.82 (0.968-3.429)			.06
		Improved	25 (23.4)		31 (31)								30 (35.7)								
		Deteriorated or unchanged	82 (76.6)		69 (69)								54 (64.3)								
	**Composite score^c^**											.15									.03
		Improved	25 (24)		31 (33)								30 (37)								
		Unchanged	64 (61.5)		53 (56.4)								45 (55.6)								
		Deteriorated	15 (14.4)		10 (10.6)								6 (7.4)								

^a^Seven missing values in the control group, 6 missing values in the intervention group in the intention-to-treat analysis, and 3 missing values in the intervention group in the per-protocol analysis.

^b^Two missing values in the intervention group in the intention-to-treat analysis, and 1 missing value in the intervention group in the per-protocol analysis.

^c^Three missing values in the control group, 8 missing values in the intervention group in the intention-to-treat analysis, and 4 missing values in the intervention group in the per-protocol analysis.

**Table 3:** Change in general self-efficacy (GSE) score from baseline to 3- and 6-month follow-ups (N=209).

			Control (n=107)		Intervention (n=102)		Odds ratio (95% CI)		*P* value		Per-protocol analysis (n=85)		Odds ratio (95% CI)		*P* value
**Three months**															
	Change in GSE score, mean (SD)	–0.038 (5.2)^a^		2.069 (5.9)^b^		N/A^c^		.03		2.557 (5.4)^d^		N/A		.01	
	Increase by ≥5 points, n (%)	11 (10.3)		23 (22.5)		2.54 (1.167-5.530)		.02		21 (24.7)		2.86 (1.293-6.342)		.01	
**Six months**															
	Change in GSE score, mean (SD)	1.380 (5.9)^a^		3.204 (6.6)^b^		N/A		.07		3.463 (6.6)^d^		N/A		.04	
	Increase by ≥5 points, n (%)	28 (26.2)		36 (35.3)		1.54 (0.851-2.782)		.15		34 (40)		1.88 (1.020-3.468)		.04	

^a^n=84 at the 3-month follow-up, and n=94 at the 6-month follow-up.

^b^n=84 at the 3-month follow-up, and n=82 at the 6-month follow-up.

^c^N/A: not applicable.

^d^n=75 at the 3- and 6-month follow-ups.

**Table 4:** Overview of self-reported sick leave at the 3- and 6-month follow-ups (N=209).

		Three months, control (n=107)^a^, n (%)	Intervention (n=102)^b^, n (%)	*P* value	Per-protocol analysis (n=85)^c^, n (%)	*P* value	Six months, control (n=107)^a^, n (%)	Intervention (n=102)^b^, n (%)	*P* value	Per-protocol analysis (n=85)^c^, n (%)	*P* value
**Level of sick leave**				.85		.76			.96		.93
	Decreased	62 (75)	65 (77)		58 (77)		82 (85)	70 (84)		64 (84)	
	Unchanged	19 (23)	16 (19)		15 (20)		11 (12)	11 (13)		11 (15)	
	Increased	2 (2)	3 (4)		2 (3)		3 (3)	2 (2)		1 (1)	

^a^n=83 at the 3-month follow-up, and n=96 at the 6-month follow-up.

^b^n=84 at the 3-month follow-up, and n=83 at the 6-month follow-up.

^c^n=75 at the 3-month follow-up, and n=76 at the 6-month follow-up.

## Discussion

### Principal Findings

There were no statistically significant differences between the control and intervention groups in the composite GSE score and level of sick leave at the 6-month follow-up. However, there was a significant difference at the 3-month follow-up. The intervention did not affect the level of sick leave, and the differences observed in the composite score were largely due to increased self-efficacy. To our knowledge, this is the first randomized controlled trial to evaluate the effects of PCC by means of a combined web-based platform and telephone support for people on sick leave due to CMDs in primary care, where most patients with CMDs are treated [9]. Modern-day mental health care should be person-centered, based on principles of partnership, and support self-management, regardless of whether care is conducted in traditional face-to-face or remote eHealth settings [37]. A strength of this study is thus the evaluation of remote PCC in addition to usual care. As this intervention was managed remotely, it was also accessible to patients, irrespective of their location. Considering the COVID-19 pandemic, this made it possible to continue the intervention without lapses or alterations. Another strength is the relatively high response rate (nearly 80%). Moreover, we see both strengths and weaknesses in using a composite score as a primary outcome. A composite score enables a mix of outcome measures with different qualities, providing a wider perspective, compared with using a single outcome measure [49]. The study participants could only be classified as improved in the composite score if they had both increased GSE and reduced level of sick leave. When interpreting our results, it is important to consider that although there was a significant effect on the composite score at the 3-month follow-up in the ITT analysis, and at the 3- and 6-month follow-ups in the ordered PP analysis, the intervention did not affect the level of sick leave, and the positive results at 3 months were driven exclusively by changes in GSE.

This study includes several limitations. First, in this study, data on sick leave and GSE were self-reported. Although self-reported sick leave data have been shown to be congruent with employers’ registers [52], not having access to complete register data impeded obtaining more detailed information on the participants’ sick leave trajectories (eg, the total number of days on sick leave throughout the intervention). Furthermore, 49% (41/83) of participants in the control group and 54% (45/84) in the intervention group reported 0% sick leave at the 3-month follow-up, indicating that the sick leave outcome had reached a floor effect already at 3 months. Consequently, the primary outcome should have been set earlier than 6 months. However, because there was no difference in sick leave levels between the groups at 3 and 6 months, either the intervention was unsuccessful in affecting the level of sick leave altogether or the effects were insignificant at these specific time points. The Swedish National Board of Health and Welfare recommends sick leave of 2 weeks for conditions such as acute stress reaction, up to 2 months for depressive episodes, and 6 months to 1 year for exhaustion disorder [53]. Thus, the decision to measure sick leave at 3 and 6 months corresponds to the estimated range of sick leave for the conditions we included in the study. In addition, 30% (29/96) of the participants in the control group and 30% (25/83) in the intervention group reported ongoing sick leave at the 6-month follow-up.

Sick leave is a complex process influenced by factors at different structural levels (eg, social insurance agencies, workplace representatives, and medical experts) [54]. Although physicians ultimately base their sick leave recommendations on the patient’s health status, the standardization of the recommendations may impede patients’ ability to exert influence on the process. Whether the participants who reported not being on sick leave at the 3-month follow-up had improved on other health-related measures such as symptom severity is unclear in the present analysis but will be a valuable subject to examine in future studies. For example, there have been discussions on whether conditions such as adjustment disorders require health care interventions or resolve on their own with time, with or without changes in self-efficacy. However, if stressors continue, these conditions are linked to decreased quality of life, risk of developing mental disorders that are more severe, and increased risk of suicide [55]. It will also be important to evaluate whether this intervention, occurring in the early stages of sick leave, will affect the sick leave process in the long term. Evidently, the CMD spectrum includes both patients who RTW after only a few weeks and those at risk of long, potentially recurrent, sick leave. Sick leave due to CMDs is the most extended among all causes of sick leave in Sweden [8]. Long-term sick leave has detrimental effects on people’s quality of life and is associated with a reduced probability of eventual RTW and subsequent economic and social deprivation [3,56]. Early interventions can play an important role in preventing a deterioration in conditions and long-term sick leave with risks of relapse [57].

Although the intervention did not lead to any difference in sick leave, it positively affected the GSE scores of the intervention group. In previous studies, higher self-efficacy was associated with accelerated RTW in patients with CMDs, especially when using self-efficacy measures specifically targeting a sense of competence toward work or RTW rather than GSE [28-32]. However, GSE aims to capture a broad and stable sense of personal competence to deal effectively with a range of stressful and challenging situations [33,50,58] and may mirror conditions where the context is complex and unpredictable and the circumstances change rapidly. As such, strengthening patients’ GSE is in line with the ambitions of PCC to improve patients’ sense of their ability to manage the situations they encounter, along with the evidence that self-efficacy is important for successful self-management [59]. The mean GSE score at baseline in our sample was lower in the control and intervention groups (approximately 25) than the mean scores from general population studies (usually approximately 29-30) [48,50,51]. The designation of a ≥5-point difference in GSE scores corresponds approximately to the reported SD in general population studies [48,50,51] and almost an SD in our sample, that is, in the scope of a minimally important change [42,43]. At 6 months, the increase in GSE in the intervention group (in both the ITT and PP analyses) resulted in an alignment of their mean GSE to the means of general populations. The GSE mean scores of the control group continued to be lower than the mean scores of both the intervention group and general population at both follow-ups. As self-efficacy is negatively associated with depression and anxiety [50,60], follow-ups assessing whether the increase in GSE in the intervention group affects mental health symptoms are justified. Such evaluations, together with a long-term assessment of the impact of the intervention on preventing future recurrences and extended sick leave, are necessary to appraise the overall value of the intervention.

### Conclusions

Our study showed that the person-centered eHealth intervention for patients on sick leave due to CMDs improved self-efficacy but did not affect sick leave levels at 3 and 6 months. Self-efficacy may have important implications for patients on sick leave due to CMDs in managing their situation. Further research is needed to evaluate effects on sick leave from a long-term perspective and verify the clinical value of these findings.

## Appendix

Multimedia Appendix 1 CONSORT-eHEALTH checklist (V 1.6.2).

## Abbreviations

CMD: common mental disorder
CONSORT-EHEALTH: Consolidated Standards of Reporting Trials of Electronic and Mobile Health Applications and Online Telehealth
GSE: general self-efficacy
GSES: General Self-Efficacy Scale
HCP: health care professional
ITT: intention-to-treat
OR: odds ratio
PCC: person-centered care
PP: per-protocol
RTW: return to work
