# Supplementary material for: Effects of a Person-Centered eHealth Intervention for Patients on Sick Leave Due to Common Mental Disorders (PROMISE Study): Open Randomized Controlled Trial
Source: JMIR Ment Health. 2022 Mar 15;9(3):e30966. doi: 10.2196/30966 (PMC8965681; doi:10.2196/30966)
Supplement: Multimedia Appendix 1 [file mental_v9i3e30966_app1.pdf]

CONSORT-EHEALTH Checklist V1.6.2 Report	Manuscript Number	30966
**(based on CONSORT-EHEALTH V1.6), available at [http://tinyurl.com/consort-ehealth-v1-6].**		
**Date completed**		
1/19/2022 8:57:30		
**by**		
Matilda Cederberg		
Effects of a person-centred eHealth intervention for patients on sick leave due to common mental disorders (PROMISE): An open randomised controlled trial		
TITLE		
**1a-i) Identify the mode of delivery in the title**		
"eHealth"		
**1a-ii) Non-web-based components or important co-interventions in title**		
**1a-iii) Primary condition or target group in the title**		
"on sick leave due to common mental disorders"		
ABSTRACT		
**1b-i) Key features/functionalities/components of the intervention and comparator in the METHODS section of the ABSTRACT**		
"the intervention group received usual care with addition of a person-centred eHealth intervention. The intervention was built on PCC principles and consisted of phone support and an interactive digital platform"		
**1b-ii) Level of human involvement in the METHODS section of the ABSTRACT**		
**1b-iii) Open vs. closed, web-based (self-assessment) vs. face-to-face assessments in the METHODS section of the ABSTRACT**		
**1b-iv) RESULTS section in abstract must contain use data**		
**1b-v) CONCLUSIONS/DISCUSSION in abstract for negative trials**		
"A person-centred eHealth intervention for patients on sick leave due to CMDs improves self-efficacy but does not affect the level of sick leave."		
INTRODUCTION		
**2a-i) Problem and the type of system/solution**		
"Because previous studies based on PCC have shown positive effects on self-efficacy and because of the potential influence of self-efficacy in affecting sick leave, it is warranted to construct and evaluate PCC interventions targeting patients on sick leave due to CMDs."		
**2a-ii) Scientific background, rationale: What is known about the (type of) system**		
"Internet interventions have proven to be a viable option to face-to-face treatments in patients with CMDs [13-16]. Due to the high accessibility and direct involvement of the patient, internet interventions may also enhance self-management [17]. To date, very few studies have evaluated interventions using eHealth alternatives for CMDs that specifically target return to work (RTW) [18]. "		
**Does your paper address CONSORT subitem 2b?**		
"Thus, this study aims to evaluate the effects of a person-centred eHealth intervention for patients on sick leave due to CMDs. "		
METHODS		
**3a) CONSORT: Description of trial design (such as parallel, factorial) including allocation ratio**		
"The study was an open randomised controlled trial (RCT) with 1:1 allocation to either a control group receiving usual care only or an intervention group receiving usual care in conjunction with a person-centred eHealth intervention embracing phone support and access to an interactive digital platform"		
**3b) CONSORT: Important changes to methods after trial commencement (such as eligibility criteria), with reasons**		
there were no changes in, for example, eligibility criteria after trial commencement		
**3b-i) Bug fixes, Downtimes, Content Changes**		
**4a) CONSORT: Eligibility criteria for participants**		
"Patients aged 18-65 years were eligible if they were currently on sick leave due to one of the following conditions in ICD-10 and diagnosed by a physician: mild to moderate depression (F32 and F33), mild to moderate anxiety disorder (F41), reaction to severe stress and adjustment disorders (F43, except post-traumatic stress disorder), which includes the Swedish diagnosis Exhaustion disorder (F43.8A). In order to reach patients early in their sick leave process, their current sick leave episode should not have exceeded 30 days. Patients were eligible only if they were employed or studying at least part-time during the past 9 months. "		
**4a-i) Computer / Internet literacy**		
**4a-ii) Open vs. closed, web-based vs. face-to-face assessments:**		
"Designated health care professionals (HCPs) consecutively screened the medical records of nine primary health care centres for eligible participants. Eligible participants were sent an information letter about the study, notifying them that further contact would be made. Next, patients were contacted by phone or they contacted the HCPs using instructions in the information letter. More information about the study was given over the phone. Patients interested in participating were sent a consent form and information about their rights as participants by regular mail. After written consent had been returned by mail to the HCPs, patients were randomised to the control or intervention group. Randomisation was based on a computer-generated random list created by a third party and stratified by age (<50 or ≥50 years) and diagnostic group (1: Depression, 2: Anxiety, 3: Stress-reactions and disorders). After randomisation, participants were informed of their study arm".		
**4a-iii) Information giving during recruitment**		
**4b) CONSORT: Settings and locations where the data were collected**		
"The study took place in a larger, socioeconomically diverse city area in Western Sweden. Nine public primary health care centres participated. The intervention and each study procedure were managed remotely"		
**4b-i) Report if outcomes were (self-)assessed through online questionnaires**		
"Data included responses to questionnaires sent by letter at baseline and after 3 and 6 months, during the intervention"		
**4b-ii) Report how institutional affiliations are displayed**		
**5) CONSORT: Describe the interventions for each group with sufficient details to allow replication, including how and when they were actually administered**		
**5-i) Mention names, credential, affiliations of the developers, sponsors, and owners**		
**5-ii) Describe the history/development process**		
**5-iii) Revisions and updating**		
**5-iv) Quality assurance methods**		
**5-v) Ensure replicability by publishing the source code, and/or providing screenshots/screen-capture video, and/or providing flowcharts of the algorithms used**		
**5-vi) Digital preservation**		
**5-vii) Access**		
"Shortly after inclusion, the HCPs called the patients to help them access the digital platform, described its features and scheduled a phone conversation. "		
**5-viii) Mode of delivery, features/functionalities/components of the intervention and comparator, and the theoretical framework**		
"In addition to usual care, the intervention group received PCC via an interactive digital platform and phone support during the 6-month intervention period... The intervention aimed to operationalise person-centred ethics into action by safeguarding the relational aspects of personhood and care according to PCC [39, 45]."..."By offering an infrastructure in the form of phone support and an interactive digital platform it was designed to facilitate the co-creation of care and work in partnership between HCPs and patients (and their extended social network if needed) without face-to-face meetings. The intervention design allowed for individual tailoring in terms of content (i.e., the personal health plans) and structure (i.e., the number, intensity and form for communication). The patients were encouraged to use the platform's different functions, but all use was optional and based on the patient's preferences."		
**5-ix) Describe use parameters**		

**5-x) Clarify the level of human involvement**		
**5-xi) Report any prompts/reminders used**		
"The patients were encouraged to use the platform's different functions, but all use was optional and based on the patient's preferences"		
**5-xii) Describe any co-interventions (incl. training/support)**		
"HCPs from different disciplines (e.g., nursing, physiotherapy, occupational therapy), conducted the intervention in a research setting separated from the primary care centres. The team of HCPs received a half-day training and education regarding symptoms, treatments, care and self-care strategies for CMDs led by psychologists and physicians specialised in stress-related mental illness. They also received an introduction to the philosophical perspective of PCC led by researchers in health and care sciences, philosophy and pedagogics. The team of HCPs also had access to a regularly held forum where they, together with specialists in PCC, could raise questions and share experiences of the practice of PCC stipulated within the context of the intervention."		
**6a) CONSORT: Completely defined pre-specified primary and secondary outcome measures, including how and when they were assessed**		
"The primary outcome of this study was a composite score of changes in GSE and sick leave at the 6-month follow-up [49]. At the 3- and 6-month follow-ups, participants in both arms were classified by the following standards:<br><br>•Participants with reduced sick leave percentage at follow-up compared to baseline and increased GSE scores by ≥5 units were classified as improved.<br>•Participants with an increased sick leave percentage at follow-up compared to baseline or reduced GSE scores by ≥5 units were classified as deteriorated.<br>•Participants who had neither deteriorated nor improved were classified as unchanged.<br><br>To calculate the primary outcome both study groups were dichotomised into two subgroups: improved and unchanged/deteriorated. The designated five-point difference corresponds closely to the reported standard deviation [50, 51] and previous research suggesting five points to be a threshold for a minimal important change [42, 43]. For further details on participants' trajectories, the composite score was also analysed without dichotomising in an ordered categorical version, including all three possible outcomes (improved/unchanged/deteriorated)."		
**6a-i) Online questionnaires: describe if they were validated for online use and apply CHERRIES items to describe how the questionnaires were designed/deployed**		
**6a-ii) Describe whether and how "use" (including intensity of use/dosage) was defined/measured/monitored**		
**6a-iii) Describe whether, how, and when qualitative feedback from participants was obtained**		
**6b) CONSORT: Any changes to trial outcomes after the trial commenced, with reasons**		
"The study took place in a larger, socioeconomically diverse city area in Western Sweden. Nine public primary health care centres participated. The intervention and each study procedure were managed remotely"		
**7a) CONSORT: How sample size was determined**		
**7a-i) Describe whether and how expected attrition was taken into account when calculating the sample size**		
**7b) CONSORT: When applicable, explanation of any interim analyses and stopping guidelines**		
"The primary outcome of this study was a composite score of changes in GSE and sick leave at the 6-month follow-up [49]. At the 3- and 6-month follow-ups, participants in both arms were classified by the following standards:<br><br>•Participants with reduced sick leave percentage at follow-up compared to baseline and increased GSE scores by ≥5 units were classified as improved.<br>•Participants with an increased sick leave percentage at follow-up compared to baseline or reduced GSE scores by ≥5 units were classified as deteriorated.<br>•Participants who had neither deteriorated nor improved were classified as unchanged.<br><br>To calculate the primary outcome both study groups were dichotomised into two subgroups: improved and unchanged/deteriorated. The designated five-point difference corresponds closely to the reported standard deviation [50, 51] and previous research suggesting five points to be a threshold for a minimal important change [42, 43]. For further details on participants' trajectories, the composite score was also analysed without dichotomising in an ordered categorical version, including all three possible outcomes (improved/unchanged/deteriorated)."		
**8a) CONSORT: Method used to generate the random allocation sequence**		
"After written consent had been returned by mail to the HCPs, patients were randomised to the control or intervention group. Randomisation was based on a computer-generated random list created by a third party and stratified by age (<50 or ≥50 years) and diagnostic group (1: Depression, 2: Anxiety, 3: Stress-reactions and disorders). After randomisation, participants were informed of their study arm. "		
**8b) CONSORT: Type of randomisation; details of any restriction (such as blocking and block size)**		
"After written consent had been returned by mail to the HCPs, patients were randomised to the control or intervention group. Randomisation was based on a computer-generated random list created by a third party and stratified by age (<50 or ≥50 years) and diagnostic group (1: Depression, 2: Anxiety, 3: Stress-reactions and disorders). After randomisation, participants were informed of their study arm. "		
**9) CONSORT: Mechanism used to implement the random allocation sequence (such as sequentially numbered containers), describing any steps taken to conceal the sequence until interventions were assigned**		
"Randomisation was based on a computer-generated random list created by a third party and stratified by age (<50 or ≥50 years) and diagnostic group (1: Depression, 2: Anxiety, 3: Stress-reactions and disorders). After randomisation, participants were informed of their study arm."		
**10) CONSORT: Who generated the random allocation sequence, who enrolled participants, and who assigned participants to interventions**		
"After written consent had been returned by mail to the HCPs, patients were randomised to the control or intervention group. Randomisation was based on a computer-generated random list created by a third party and stratified by age (<50 or ≥50 years) and diagnostic group (1: Depression, 2: Anxiety, 3: Stress-reactions and disorders). After randomisation, participants were informed of their study arm."		
**11a) CONSORT: Blinding - If done, who was blinded after assignment to interventions (for example, participants, care providers, those assessing outcomes) and how**		
**11a-i) Specify who was blinded, and who wasn't**		
" After randomisation, participants were informed of their study arm."		
**11a-ii) Discuss e.g., whether participants knew which intervention was the "intervention of interest" and which one was the "comparator"**		
**11b) CONSORT: If relevant, description of the similarity of interventions**		
"Patients on sick leave for CMDs are usually offered an appointment with a physician to follow up on sick leave and make treatment decisions. Treatment may consist of medication or psychological therapies such as cognitive behaviour therapy [10]. Depending on the services available at each primary health care centre, usual care can also include contact with a physiotherapist, rehabilitation coordinator or occupational therapist, as well as group sessions targeting specific symptoms or problems. "		
**12a) CONSORT: Statistical methods used to compare groups for primary and secondary outcomes**		
"Descriptive statistics were used to characterise the study groups. Between-group differences in baseline characteristics were analysed using Pearson´s chi-squared test for categorical variables, Fisher´s exact test for dichotomous variables and Student´s t-test (independent) for continuous variables. Between-group differences in the dichotomous version of the composite score and differences in improvement of ≥5 units on the GSES were tested using Fisher´s exact test. Binary logistic regression was used to calculate odds ratios (ORs) with 95% confidence intervals (CIs). The Mantel-Haenszel chi-square test was applied for the ordered (three levels) categorical version of the composite score and self-reported sick leave. Between-group differences in changes in GSE scores were analysed using the Mann-Whitney U test. "		
**12a-i) Imputation techniques to deal with attrition / missing values**		
"Missing outcome data for GSE in the composite score at the 3- and 6-month follow-ups were imputed using the last observation value carried forward. "..." Intention-to-treat (ITT) and per-protocol (PP) analysis were performed. The PP analysis included intervention participants who had at least one phone conversation with the HCPs leading to a health plan and who had used the digital platform at least once during the intervention period."		
**12b) CONSORT: Methods for additional analyses, such as subgroup analyses and adjusted analyses**		
"Sensitivity analysis were performed to assure robustness by excluding patients who reported other causes of sick leave than CMDs at follow-up (n=3) and participants who reported 0% sick leave at baseline (n=6). The six participants who reported 0% sick leave at baseline were on sick leave when they gave oral consent to participate, but their sick leave periods had expired once they were randomised. IIntention-to-treat (ITT) and per-protocol (PP) analysis were performed. The PP analysis included intervention participants who had at least one phone conversation with the HCPs leading to a health plan and who had used the digital platform at least once during the intervention period."		
RESULTS		
**13a) CONSORT: For each group, the numbers of participants who were randomly assigned, received intended treatment, and were analysed for the primary outcome**		
"Of 215 participants in total, 108 were randomised to the control group and 107 to the intervention group. After one participant in the control group and five participants in the intervention group withdrew consent, the final sample included 209 participants; 107 in the control group and 102 in the intervention group."		
**13b) CONSORT: For each group, losses and exclusions after randomisation, together with reasons**		
Yes, this is shown in a CONSORT diagram		
**13b-i) Attrition diagram**		

**14a) CONSORT: Dates defining the periods of recruitment and follow-up**		
"Participant recruitment lasted from February 2018 to June 2020."		
**14a-i) Indicate if critical "secular events" fell into the study period**		
**14b) CONSORT: Why the trial ended or was stopped (early)**		
Not applicable, the trial stopped when all participants needed were included		
**15) CONSORT: A table showing baseline demographic and clinical characteristics for each group**		
Presented in table 1 in the manuscript		
**15-i) Report demographics associated with digital divide issues**		
Presented in table 1 in the manuscript		
**16a) CONSORT: For each group, number of participants (denominator) included in each analysis and whether the analysis was by original assigned groups**		
**16-i) Report multiple "denominators" and provide definitions**		
"To achieve a power of 80% based on an alpha error of 0.05 a minimum of 91 participants was required in each study group to detect an improvement in the composite score (20% in the control group and 40% in the intervention group)."		
**16-ii) Primary analysis should be intent-to-treat**		
**17a) CONSORT: For each primary and secondary outcome, results for each group, and the estimated effect size and its precision (such as 95% confidence interval)**		
"Between-group differences in the dichotomous version of the composite score and differences in improvement of ≥5 units on the GSES were tested using Fisher´s exact test. "..."The significance level was set at P<0.05 (two-sided)."		
**17a-i) Presentation of process outcomes such as metrics of use and intensity of use**		
**17b) CONSORT: For binary outcomes, presentation of both absolute and relative effect sizes is recommended**		
"Binary logistic regression was used to calculate odds ratios (ORs) with 95% confidence intervals (CIs)."		
**18) CONSORT: Results of any other analyses performed, including subgroup analyses and adjusted analyses, distinguishing pre-specified from exploratory**		
"Missing outcome data for GSE in the composite score at the 3- and 6-month follow-ups were imputed using the last observation value carried forward. Sensitivity analysis were performed to assure robustness. Intention-to-treat (ITT) and per-protocol (PP) analysis were performed."		
**18-i) Subgroup analysis of comparing only users**		
**19) CONSORT: All important harms or unintended effects in each group**		
Not applicable as we in this study do not address unintended effects or potential harms. However that will be part of an upcoming process evaluation.		
**19-i) Include privacy breaches, technical problems**		
**19-ii) Include qualitative feedback from participants or observations from staff/researchers**		
DISCUSSION		
**20) CONSORT: Trial limitations, addressing sources of potential bias, imprecision, multiplicity of analyses**		
**20-i) Typical limitations in ehealth trials**		
"This study has several limitations. First, in this study data on sick leave and GSE were self-reported. Although self-reported sick leave data have shown to be congruent with employers' register [52], not having access to complete register data impeded more detailed information on the participants' sick leave trajectories (e.g., the total number of days on sick leave throughout the intervention). Furthermore, about 50% of the participants in both study arms reported 0% sick leave at the 3-month-follow up, indicating that the sick leave outcome reached a floor effect already at 3 months. Consequently, the primary outcome should have been set earlier than 6 months. However, because there was no difference in sick leave levels between the groups at 3-and 6-months, either the intervention was unsuccessful in affecting the level of sick leave altogether, or the effects were insignificant at these specific time points"		
**21) CONSORT: Generalisability (external validity, applicability) of the trial findings**		
**21-i) Generalizability to other populations**		
**21-ii) Discuss if there were elements in the RCT that would be different in a routine application setting**		
**22) CONSORT: Interpretation consistent with results, balancing benefits and harms, and considering other relevant evidence**		
**22-i) Restate study questions and summarize the answers suggested by the data, starting with primary outcomes and process outcomes (use)**		
"No statistically significant differences between the control and intervention group on the composite score of self-efficacy and level of sick leave at the 6-month follow-up. However, there was a significant difference at the 3-month follow-up. The intervention did not affect the level of sick leave and the differences observed in the composite score were largely due to increased self-efficacy"		
**22-ii) Highlight unanswered new questions, suggest future research**		
Other information		
**23) CONSORT: Registration number and name of trial registry**		
" The trial was registered in the ClinicalTrials.gov (Identifier NCT03404583)"		
**24) CONSORT: Where the full trial protocol can be accessed, if available**		
"A detailed description of the intervention has been published elsewhere [44]."		
**25) CONSORT: Sources of funding and other support (such as supply of drugs), role of funders**		
"This work was supported by The Swedish Research Council for Health, Working Life and Welfare (reference number 2016-07418, 2017-00557 and 2019-01726). The funder has no role in the design of the study, data collection, analysis, or interpretation. The study was financed by grants from the Swedish state under the agreement between the Swedish government and the country councils, the ALF agreement (ALFGBG-772191 and ALFGBG-932659). "		
**X26-i) Comment on ethics committee approval**		
**x26-ii) Outline informed consent procedures**		
**X26-iii) Safety and security procedures**		
**X27-i) State the relation of the study team towards the system being evaluated**		
